# Pandemrix® vaccination is not associated with increased risk of islet autoimmunity or type 1 diabetes in the TEDDY study children

**DOI:** 10.1007/s00125-017-4448-3

**Published:** 2017-10-09

**Authors:** Helena Elding Larsson, Kristian F. Lynch, Maria Lönnrot, Michael J. Haller, Åke Lernmark, William A. Hagopian, Jin-Xiong She, Olli Simell, Jorma Toppari, Anette-G. Ziegler, Beena Akolkar, Jeffrey P. Krischer, Marian J. Rewers, Heikki Hyöty, for the TEDDY Study Group

**Affiliations:** 1 Department of Clinical Sciences Malmö, Lund University CRC, Skåne University Hospital SUS, Jan Waldenströms gata 35; 60:11, 20502 Malmö, Sweden; 2 Health Informatics Institute, Morsani College of Medicine, University of South Florida, Tampa, FL USA; 3 Faculty of Medicine and Life Sciences, University of Tampere, Tampere, Finland; 4 Department of Dermatology, Tampere University Hospital, Tampere, Finland; 5 Department of Pediatrics, University of Florida, Gainesville, FL USA; 6 Pacific Northwest Diabetes Research Institute, Seattle, WA USA; 7 Center for Biotechnology and Genomic Medicine, Medical College of Georgia, Augusta University, Augusta, GA USA; 8 Department of Pediatrics, Turku University Hospital, Turku, Finland; 9 Department of Physiology, Institute of Biomedicine, University of Turku, Turku, Finland; 10 Institute of Diabetes Research, Helmholtz Zentrum München, and Klinikum rechts der Isar, Technische Universität München, Munich, Germany; 11 Forschergruppe Diabetes e.V., Neuherberg, Germany; 12 National Institute of Diabetes & Digestive & Kidney Diseases, Bethesda, MD USA; 13 Barbara Davis Center for Childhood Diabetes, University of Colorado, Aurora, CO USA; 14 Fimlab Laboratories, Pirkanmaa Hospital District, Tampere, Finland

**Keywords:** Influenza vaccine, Islet autoimmunity, Pandemrix, Squalene, Swine flu, Type 1 diabetes, Vaccination

## Abstract

**Aims/hypothesis:**

During the A/H1N1 2009 (A/California/04/2009) pandemic, mass vaccination with a squalene-containing vaccine, Pandemrix®, was performed in Sweden and Finland. The vaccination was found to cause narcolepsy in children and young adults with the *HLA-DQ 6.2* haplotype. The aim of this study was to investigate if exposure to Pandemrix® similarly increased the risk of islet autoimmunity or type 1 diabetes.

**Methods:**

In The Environmental Determinants of Diabetes in the Young (TEDDY) study, children are followed prospectively for the development of islet autoimmunity and type 1 diabetes. In October 2009, when the mass vaccination began, 3401 children at risk for islet autoimmunity and type 1 diabetes were followed in Sweden and Finland. Vaccinations were recorded and autoantibodies against insulin, GAD65 and insulinoma-associated protein 2 were ascertained quarterly before the age of 4 years and semi-annually thereafter.

**Results:**

By 5 August 2010, 2413 of the 3401 (71%) children observed as at risk for an islet autoantibody or type 1 diabetes on 1 October 2009 had been vaccinated with Pandemrix®. By 31 July 2016, 232 children had at least one islet autoantibody before 10 years of age, 148 had multiple islet autoantibodies and 96 had developed type 1 diabetes. The risk of islet autoimmunity was not increased among vaccinated children. The HR (95% CI) for the appearance of at least one islet autoantibody was 0.75 (0.55, 1.03), at least two autoantibodies was 0.85 (0.57, 1.26) and type 1 diabetes was 0.67 (0.42, 1.07). In Finland, but not in Sweden, vaccinated children had a lower risk of islet autoimmunity (0.47 [0.29, 0.75]), multiple autoantibodies (0.50 [0.28, 0.90]) and type 1 diabetes (0.38 [0.20, 0.72]) compared with those who did not receive Pandemrix®. The analyses were adjusted for confounding factors.

**Conclusions/interpretation:**

Children with an increased genetic risk for type 1 diabetes who received the Pandemrix® vaccine during the A/H1N1 2009 pandemic had no increased risk of islet autoimmunity, multiple islet autoantibodies or type 1 diabetes. In Finland, the vaccine was associated with a reduced risk of islet autoimmunity and type 1 diabetes.

**Electronic supplementary material:**

The online version of this article (10.1007/s00125-017-4448-3) contains peer-reviewed but unedited supplementary material, which is available to authorised users.

## Introduction

Clinical diagnosis of type 1 diabetes is preceded by an autoimmune destructive process against the pancreatic islet beta cells; a prodromal period that may last a few months or several years. The risk of developing autoimmunity in genetically susceptible children is widely considered to be increased by perinatal or early childhood environmental exposures [1]. While genetic risk factors, such as *HLA-DR-DQ* [2] and non-HLA genetic factors [3], have been implicated, the environmental conditioning or trigger exposures have not yet been defined. In The Environmental Determinants of Diabetes in the Young (TEDDY) study, we have the opportunity to analyse triggers in relation to seroconversion to islet autoimmunity as a first primary endpoint, as over 70% of children with multiple islet autoantibodies develop type 1 diabetes over a 10-year period [4]. It has been speculated that vaccinations early in life may alter the immune response to infections, leading to a disturbed capacity to distinguish between self and non-self and thereby increasing the risk of autoimmune reactions. However, previous studies do not support the notion that type 1 diabetes can be triggered by vaccinations [5, 6].

During the H1N1 influenza pandemic in 2009–2010, mass vaccination of both children and adults took place in many countries. In Sweden and Finland, Pandemrix®, a vaccine containing the squalene-based adjuvant ASO3, was used while other countries used other types of vaccine. A few months after the Pandemrix® vaccination programme, the incidence of new narcolepsy diagnoses increased sharply in both countries. Additional investigations seeking an understanding of the potential mechanisms associated with Pandemrix® and narcolepsy suggested that the Pandemrix® vaccination might have resulted in the loss of orexin-producing neurons, leading to the development of narcolepsy in these individuals [7–9]. The mechanism that mediates this effect is not fully understood, but it seems that both the composite influenza virus vaccine and the squalene adjuvant could contribute to the induction of orexin-specific autoimmunity [10].

The aim of the present study was to investigate if exposure to the Pandemrix® vaccine affected the incidence of islet autoimmunity and type 1 diabetes in genetically susceptible children. This was an observational study carried out among high-risk children followed prospectively from birth [11].

## Methods

### Participants

Participants were included in the TEDDY study, a prospective cohort study funded by the National Institutes of Health with the primary goal to identify environmental causes of type 1 diabetes [11]. The association between the Pandemrix® vaccination and the risk of narcolepsy was initially found in Finland and later in Sweden, representing two of the TEDDY countries. The other countries included in TEDDY are the USA (with three centres in Colorado, Georgia/Florida and Washington) and one additional centre in Europe (Germany). Although the same protocol applied to all TEDDY centres [11], the current analyses only include children from Sweden and Finland as Pandemrix® was exclusively, and with a high coverage, administered in those TEDDY countries.

At all TEDDY sites, children (*n* = 440,000) representing both the general population and first-degree relatives to individuals with type 1 diabetes were screened at birth during the period 1 September 2004 to 1 March 2010 for genetic type 1 diabetes risk, defined by HLA genotype, as described previously [12, 13]. A high-risk population of 8676 children was recruited for follow-up from 3 months of age. During the first 4 years, all children were examined every third month. Thereafter and currently, children still at risk for islet autoimmunity are being followed biannually until the age of 15 years [11]. Children with one or several islet autoantibodies continue monitoring on a 3-month schedule after 4 years of age. All participants and their legal representatives have given informed consent to participate in the study. The regional ethics committees in all participating countries approved the study.

**Fig. 1:**
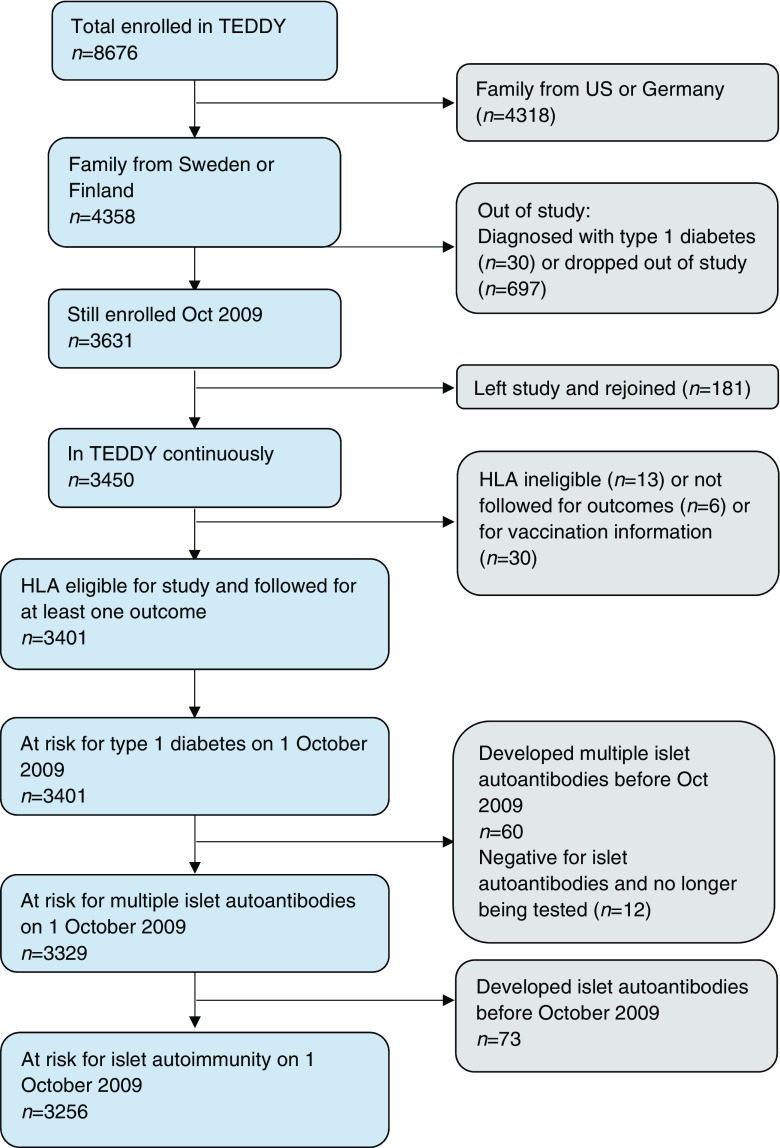
Flow chart of the study population

The mass vaccination period in both Sweden and Finland was during the winter of 2009–2010 (1 October to 31 March). On 1 October 2009, 727/4358 (17%) children were no longer considered at risk for type 1 diabetes as they had either developed the disease (*n* = 30) or dropped out of the study (*n* = 697) and were therefore excluded from the analyses. An additional 181 children were excluded for leaving the TEDDY study temporarily and re-joining after the mass vaccination, 13 were excluded for being HLA ineligible and 36 children were excluded as they either failed to have study outcomes measured (*n* = 6) or vaccination data collected (*n* = 30) after the mass vaccination. Of the remaining 3401 children still considered at risk of developing type 1 diabetes on 1 October 2009, 3256 had not yet developed islet autoimmunity and 3329 had not developed multiple islet autoantibodies (Fig. 1).

### Autoantibodies and type 1 diabetes

The primary outcome in the TEDDY study is the development of persistent confirmed autoantibodies to glutamic acid decarboxylase (GADA), insulinoma-associated protein 2 (IA-2A) or insulin (IAA), measured at all study visits and analysed by radiobinding assays [14, 15]. All samples were initially analysed at the reference laboratory at the University of Bristol, UK. Islet autoimmunity was defined as persistent (at least two consecutive visits) presence of one or more of these autoantibodies, confirmed in the second reference laboratory at the Barbara Davis Center for Childhood Diabetes (University of Colorado, Denver, CO, USA) and multiple islet autoantibodies was defined as more than one persistent confirmed autoantibody. Type 1 diabetes was diagnosed using the ADA criteria [16].

### Vaccination data

At all TEDDY clinic visits, the parents were asked to report if the child had been given any vaccination since the last visit. Type, dose and date of vaccination was recorded by a TEDDY nurse. If possible, the vaccination was verified by checking the child’s vaccination card. The mass vaccination with Pandemrix® began on 1 October 2009. Among the 3401 children considered at risk for type 1 diabetes on this date, 2413 had a recorded Pandemrix® vaccination by 5 August 2010. If more than one dose was received, the date of first vaccination was included in the analyses.

### Statistical methods

Children were followed for outcomes from 1 October 2009. For children born between 1 October 2009 and 1 March 2010 the follow-up was from birth. Only 26 children born after the mass vaccination had begun were vaccinated for H1N1. Differences in cumulative incidence of islet autoantibodies, multiple islet autoantibodies and type 1 diabetes between vaccination groups were examined in Kaplan–Meier analyses. For children who were vaccinated, survival time was from date of first vaccination. For children who were not vaccinated for H1N1, survival time was from 1 October 2009. Time-dependent Cox proportional hazard models were used to explore if vaccination for H1N1 modified the risk of type 1 diabetes outcomes. In the Cox models, the observations were left truncated before 1 October 2009 and right censored after 31 July 2016 or after the age of 10 years. All models were adjusted for country of residence, the presence of a first-degree relative with type 1 diabetes, HLA, sex and age of child on 1 March 2010. Final models were also adjusted for other genetic type 1 diabetes risk factors (*INS*-23Hph1 [rs689], *PTPN22* R620W [rs2476601], *CTLA4* T17A [rs231775], and SNPs rs2292239 in *ERBB3*, rs3184504 in *SH2B3*, rs10517086 and rs12708716 in *CLEC16A*, rs4948088 in *COBL*), maternal education at 9 months of age and probiotic use before 90 days of age. Maternal education was categorised as primary education (*n* = 764), some college/trade school (*n* = 719), college degree (*n* = 1851) or missing education (*n* = 67). In a competing risk analysis, we explored the relationship between Pandemrix® vaccination and the cause-specific risk of IAA or GADA as the first solitary appearing islet autoantibody [17]. In each model, children who seroconverted for a competing islet autoantibody that was not of interest were censored after the day of seroconversion. The final models were also examined by age of child (< 2 and ≥ 2 years) as the incidence of IAA and GADA as the first appearing islet autoantibody is known to differ considerably before and after 2 years of age [17]. The association between Pandemrix® vaccine (yes, no) and type 1 diabetes outcomes were reported as HRs with 95% CIs. *p* values < 0.05 were considered statistically significant. To examine for increased risk of type 1 diabetes outcomes within subgroups, interactions were tested between whether or not the child had received the H1N1 vaccine and HLA, sex, country of residence, age or family history of type 1 diabetes. The interactions were considered descriptive and secondary and a *p* value < 0.05 was considered an important interaction to investigate further. Statistical analysis was performed using SAS version 90.4 (SAS Institute, Cary, NC, USA).

## Results

**Fig. 2:**
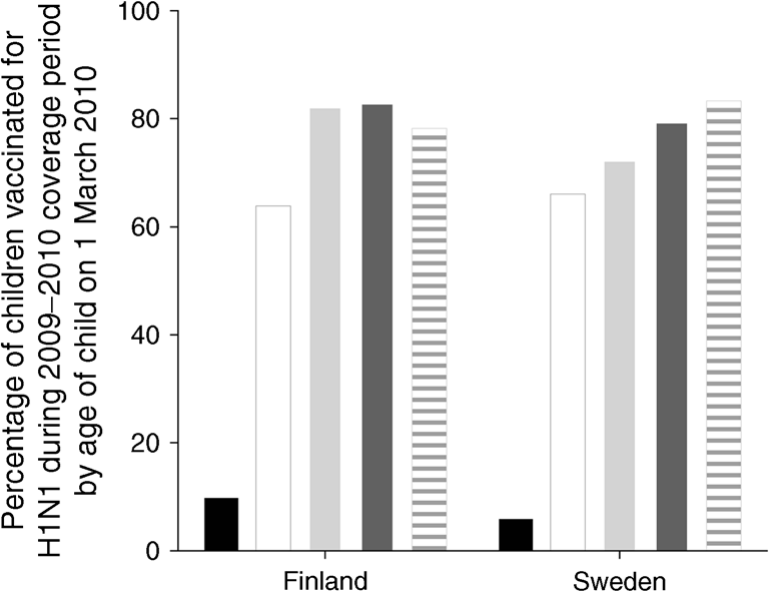
H1N1 coverage as of 1 March 2010 among children born in Finland and Sweden; coverage shown for children aged < 6 months (solid black bars), 6–11 months (solid white bars), 12–23 months (solid light grey bars), 24–36 months (solid dark grey bars) and ≥ 36 months (light grey striped bars)

Of the 3401 children considered at risk for type 1 diabetes as of 1 October 2009, 2413 (70.9%) were vaccinated with Pandemrix®. Of the children vaccinated, 98.8% (2385/2413) received their first vaccination between 1 October 2009 and 31 March 2010, five had their first vaccination in September 2009 and 22 had their vaccination between 1 April and 5 August 2010. Of the children vaccinated in Sweden, 72.9% (1010/1385) received a second Pandemrix® vaccination within a median 2.0 (interquartile range 1.5–3.0) months; however, only 0.6% (6/1028) of children from Finland received a second vaccination. The vaccination coverage by country and age of the child on 1 March 2010 (all children born) is shown in Fig. 2. In both Finland and Sweden, the coverage was low before the age of 6 months (all *p* < 0.001) and children were more likely to receive the vaccine if the mother had a college degree when the child was 9 months of age (see electronic supplementary material Table 1).

At the time of the current analyses (31 July 2016), 232 children had developed persistent confirmed islet autoantibodies by 10 years of age (135 in Sweden and 97 in Finland), 148 children had developed multiple islet autoantibodies (81 in Sweden and 67 in Finland) and 96 had developed type 1 diabetes (47 in Sweden and 49 in Finland) (Table 1).

**Table 1:** Proportional hazards models of HIN1 vaccination (yes vs no) on risk of islet autoantibodies, multiple islet autoantibodies and type 1 diabetes before age 10 years adjusting for factors in table and also stratified by the factor subgroups

Factor and group	Total (*N* ^a^)	H1N1 vaccination in relation to risk of islet autoantibodies			H1N1 vaccination in relation to risk of multiple islet autoantibodies			H1N1 vaccination in relation to risk of type 1 diabetes		
		Events (*n*)	HR (95% CI)	*p* value^b^	Events (*n*)	HR (95% CI)	*p* value^b^	Events (*n*)	HR (95% CI)	*p* value^b^
All children	3401	232	0.76 (0.57, 1.02)		148	0.92 (0.63, 1.35)		96	0.68 (0.43, 1.06)	
Country										
Finland	1438	97	0.50 (0.32, 0.78)		67	0.56 (0.33, 0.96)		49	0.41 (0.23, 0.76)	
Sweden	1963	135	1.01 (0.68, 1.50)	0.008	81	1.41 (0.82, 2.46)	0.01	47	1.10 (0.53, 2.24)	0.02
Sex										
Female	1659	103	0.91 (0.58, 1.43)		63	0.80 (0.45, 1.43)		46	0.60 (0.31, 1.16)	
Male	1742	129	0.67 (0.46, 0.99)	0.46	85	1.03 (0.62, 1.72)	0.43	50	0.77 (0.41, 1.45)	0.35
Family history										
General population	3123	199	0.69 (0.50, 0.94)		122	0.83 (0.55, 1.26)		78	0.64 (0.39, 1.06)	
First-degree relative	278	33	1.30 (0.54, 3.13)	0.12	26	1.66 (0.59, 4.68)	0.15	18	0.71 (0.24, 2.07)	0.65
*HLA-DR*										
*DR-4/4*	665	41	0.85 (0.41, 1.78)		27	1.40 (0.52, 3.74)		20	0.55 (0.20, 1.49)	
*DR-4/8*	695	47	0.61 (0.32, 1.17)		25	0.62 (0.25, 1.56)		13	0.13 (0.04, 0.49)	
*DR-3/4*	1298	111	0.72 (0.47, 1.10)		80	0.76 (0.46, 1.26)		53	0.74 (0.40, 1.38)	
*DR-3/3*	649	28	0.77 (0.32, 1.87)	0.68	12	1.73 (0.41, 7.30)	0.32	7	7.74 (0.80, 74.7)	0.14
Age on 1 March 2010										
< 1 year	752	71	0.76 (0.46, 1.25)		44	0.75 (0.40, 1.39)		26	0.26 (0.10, 0.70)	
1 year	646	50	1.13 (0.55, 2.33)		33	1.52 (0.58, 4.01)		16	2.00 (0.45, 8.97)	
2 year	649	43	0.75 (0.36, 1.58)		25	0.53 (0.20, 1.39)		23	0.70 (0.26, 1.91)	
3 year	587	34	0.76 (0.34, 1.70)		23	1.07 (0.36, 3.17)		17	0.61 (0.21, 1.83)	
≥ 4 years	722	34	0.57 (0.26, 1.24)	0.73	23	2.48 (0.57, 10.9)	0.07	14	1.24 (0.27, 5.71)	0.19

^a^Total number of children on 1 October 2009 who were observed at risk of type 1 diabetes; 145 were no longer observed at risk of islet autoimmunity, and 72 no longer at risk of multiple islet autoantibodies

^b^ *p* value test of multiplicative interaction between subgroups; all HRs are adjusted for factors in table

There was no increased risk of any islet autoantibody (HR 0.76 [95% CI 0.57, 1.02]), multiple islet autoantibodies (HR 0.92 [95% CI 0.63, 1.35]) or type 1 diabetes (HR 0.68 [95% CI 0.43, 1.06]) among the vaccinated children. In contrast, the risk of any islet autoantibody (HR 0.50 [95% CI 0.32, 0.78]), multiple islet autoantibodies (HR 0.56 [95% CI 0.33, 0.96]) and type 1 diabetes (HR 0.41 [95% CI 0.23, 0.76]) was decreased among vaccinated children in Finland, but this was not seen in Sweden (HR 1.01 [95% CI 0.68, 1.50], HR 1.41 [95% CI 0.82, 2.46] and HR 1.10 [95% CI 0.53, 2.24], respectively) (Table 1). HLA genotype, age at vaccination, sex or family history of type 1 diabetes did not modify the association between H1N1 vaccination and outcomes (Table 1).

**Fig. 3:**
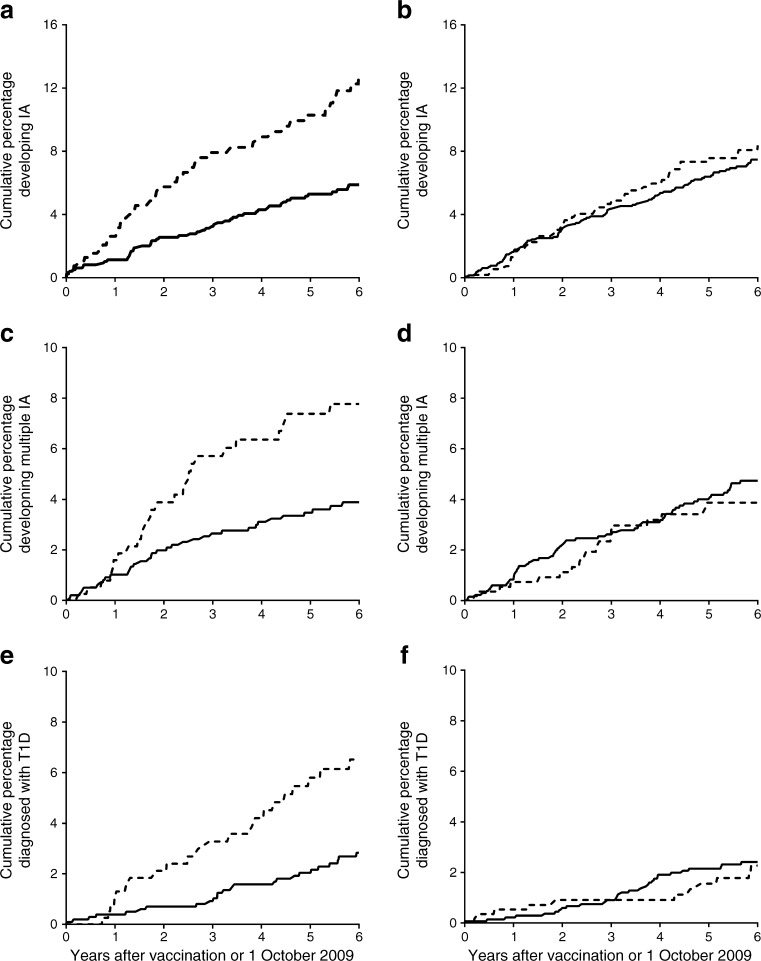
Kaplan–Meier curves showing the cumulative percentage of vaccinated (solid line) and unvaccinated (dashed line) children developing islet autoantibodies (IA) in (**a**) Finland with an average follow-up time of 5.3 years for vaccinated (*n* = 976) and 4.7 years for unvaccinated (*n* = 397) children and (**b**) Sweden with an average follow-up time of 5.4 years for vaccinated (*n* = 1309) and 5.0 years for unvaccinated (*n* = 563) children; multiple islet autoantibodies in (**c**) Finland with an average follow-up time of 5.3 years for vaccinated (*n* = 1000) and 4.8 years for unvaccinated (*n* = 400) children and (**d**) Sweden with an average follow-up time of 5.5 years for vaccinated (*n* = 1347) and 5.2 years for unvaccinated (*n* = 569) children. Type 1 diabetes (T1D) in (**e**) Finland with an average follow-up time of 5.5 years for vaccinated (*n* = 1027) and 5.1 years for unvaccinated (*n* = 410) children and (**f**) Sweden with an average follow-up time of 5.7 years for vaccinated (*n* = 1384) and 5.4 years for unvaccinated (*n* = 578) children, after the child was vaccinated or after 1 October 2009 for the children who were not vaccinated

The cumulative incidence of each of the outcomes in the two countries after vaccination or while considered at risk after 1 October 2009 is shown in Fig. 3. In Finland, the decreased risk of islet autoimmunity and multiple islet autoantibodies for the vaccinated children was seen primarily between 6 months and 36 months after the first vaccination. The cause-specific risk of IAA as a first solitary autoantibody, before other islet autoantibodies appear, primarily occurred in younger children and tended to be more frequent in Finland than in Sweden [17]. Therefore, we examined whether the incidence of IAA or GADA as first appearing solitary autoantibody separately, or any islet autoantibody, in Finland and Sweden, in both younger and older children shortly after vaccination were influenced by the vaccination after adjusting for previously reported risk factors. Genetic risk factors previously found to be associated with islet autoimmunity, including *INS*-23Hph1 (rs689), *PTPN22* R620W (rs2476601), *CTLA4*, T17A (rs231775), SNPs rs2292239 in *ERBB3*, rs3184504 in *SH2B3*, rs10517086 and rs12708716 in *CLEC16A*, and rs4948088 in *COBL* [18] were included in this analysis, as well as probiotic use before 90 days of age [19], maternal age at birth of child and maternal education (Table 2). After adjustment, the HR for islet autoantibodies (0.47 [95% CI 0.29, 0.75]), for multiple islet autoantibodies (0.50 [95% CI 0.28, 0.90]) and for type 1 diabetes (0.38 [95% CI 0.20, 0.72]) remained significantly decreased in Finland. Moreover, cause-specific risk of IAA as the first appearing islet autoantibody was decreased, particularly among children vaccinated at younger than 2 years of age (Table 2).

**Table 2:** Association between H1N1 vaccination and type 1 diabetes outcomes before age 10 years adjusting for sex, family history of type 1 diabetes, probiotics before 90 days, maternal education, maternal age, *HLA-DR-DQ* high-risk genotypes, *INS*-23Hph1 (rs689), *PTPN22* R620W (rs2476601), *CTLA4* T17A (rs231775), SNPs rs2292239 in *ERBB3*, rs3184504 in *SH2B3*, rs10517086 and rs12708716 in *CLEC16A*, and rs4948088 in *COBL*

Variable	Multiple proportional hazard model of H1N1 on type 1 diabetes outcomes									
	First appearing islet autoantibodies						Multiple islet autoantibodies		Type 1 diabetes	
	IAA		GADA		Any					
	HR	*p* value	HR	*p* value	HR	*p* value	HR	*p* value	HR	*p* value
Adjusted^a^	0.64	0.10	0.78	0.29	0.75	0.07	0.85	0.41	0.67	0.09
Finland										
Adjusted^a^	0.38	0.01	0.55	0.12	0.47	0.002	0.50	0.02	0.38	0.003
< 2 years^b^	0.30	0.009	1.18	0.79	0.51	0.03	0.45	0.04	0.25	0.002
≥ 2 years^b^	0.52	0.35	0.51	0.14	0.51	0.06	0.58	0.21	0.62	0.34
Sweden										
Adjusted^a^	0.88	0.70	0.98	0.96	1.03	0.89	1.33	0.34	1.19	0.67
< 2 years^b^	0.79	0.57	1.08	0.87	0.96	0.88	1.31	0.46	1.81	0.33
≥ 2 years^b^	0.32	0.15	1.12	0.81	0.97	0.94	1.33	0.57	0.82	0.70

^a^Variable included in multivariate proportional hazards model; all variables available on 2966 children at risk of islet autoantibodies, of which 214 developed islet autoantibodies; 3037 observed at risk for multiple islet autoantibodies, of which 140 developed multiple islet autoantibodies; and 3103 observed at risk for type 1 diabetes, of which 91 developed type 1 diabetes

^b^Age of child on 1 March 2010

In Sweden, 1.2% of children observed at risk for type 1 diabetes were vaccinated with the seasonal flu vaccine before (0.2%) or only after (1.0%) the mass vaccination for H1N1 had begun. The frequency of receiving the seasonal flu vaccine was similar between children who were vaccinated for H1N1 (1.3%) compared with children who were not (1.0%). In Finland, 58.7% of children who were vaccinated for H1N1 had a seasonal flu vaccine either before (36.1%) or only after (22.6%) the H1N1 vaccination. This was significantly higher compared with the children who were not vaccinated for H1N1 (28.3% received seasonal flu vaccine,11.7% before [*p* < 0.001] and 16.6% only after [*p* ≤ 0.01]). Mothers could also have received the seasonal flu vaccine during pregnancy; however, the percentage was similar between countries (Finland, 2.1%; Sweden 1.4%). Interestingly, of the 1.7% (58/3381) of mothers who did get the seasonal flu vaccine during pregnancy, 6.2% (46/743) were mothers of younger children (aged < 1 year on 1 March 2010) and the vaccine was given towards the end of the mass vaccination. Of these mothers, only 2/46 had their child vaccinated for H1N1. Seasonal flu vaccine administered to mothers during pregnancy or children during follow-up did not explain the association between H1N1 vaccination and type 1 diabetes outcomes. The seasonal flu vaccine did not have a modifying effect on the association between H1N1 vaccination and type 1 diabetes outcomes (data not shown). Furthermore, in Sweden, a second vaccination dose did not influence any of the type 1 diabetes outcomes (data not shown).

When analysing these results over time, we did not find any short- or long-term initiating or accelerating effects of the Pandemrix® vaccination on risk of islet autoimmunity, multiple islet autoantibodies or type 1 diabetes.

## Discussion

In this study, we investigated whether risk of islet autoimmunity and type 1 diabetes is increased in children who have been vaccinated with the squalene (ASO3)-containing H1N1 flu vaccine (Pandemrix®). Notably, Pandemrix® has been associated with autoimmunity to orexin-producing cells and the development of narcolepsy. As such, we hypothesised that this vaccine may not only promote autoimmunity to orexin-producing cells but also islet autoantigens. The results of our study do not support this hypothesis and argue against any connection between the H1N1 mass vaccination campaign and the risk of type 1 diabetes. In fact, we found that the incidence of type 1 diabetes outcomes in Finland was actually lower in children vaccinated against H1N1 than in non-vaccinated children. Theoretically, this finding could indicate that the vaccine may have reduced the risk of type 1 diabetes in higher risk populations by unknown mechanisms.

In the prospective cohort of children followed in the TEDDY study, we have the unique capacity to analyse environmental factors associated with the initiation of islet autoimmunity and development of type 1 diabetes. Type 1 diabetes is preceded by progressive autoimmune destruction of beta cells, which can last just a few months or up to many years. Since islet autoimmunity and multiple islet autoantibodies are known to confer over 70% risk to the development of type 1 diabetes within 10 years [4], it was important to consider these markers as surrogate endpoints when analysing a hypothetical increased risk of the disease during the first few years after the vaccination. By examining for risk of seroconversion to two or more islet autoantibodies as an endpoint along with type 1 diabetes, we have enabled an earlier detection of an increased or decreased risk of clinical type 1 diabetes.

Among a wide range of data currently being collected in TEDDY, vaccination records are prospectively documented in the study. Pandemrix® was used exclusively as the H1N1 vaccine in Sweden and Finland. Other types of vaccine were used in the USA as part of the H1N1 vaccination programme. In Germany, Pandemrix® was used as part of the vaccination programme, but it had low coverage. Due to heterogeneity and low numbers, we did not include the German TEDDY population in this analysis. Therefore, these analyses were limited to Swedish and Finnish children. A weakness in our study is that our population was selected for HLA genotypes associated with risk for type 1 diabetes [12]. One cannot exclude the possibility that the vaccine may increase the risk of islet autoimmunity in children with lower risk genotypes, who were screened but not enrolled for follow-up in TEDDY. This may be further emphasised by the observation that all children who developed narcolepsy after the Pandemrix® vaccination had HLA genotypes containing HLA-DQB1*06:02 [10]. Because the *DQ-0602* genotype is actually protective against the development of type 1 diabetes, children with this genotype were excluded from the TEDDY study. As HLA plays such a critical role in the presentation of antigens to the immune system and how the immune system reacts immunologically to infections, individuals with different HLA genotypes are known to react differentially to vaccinations [20].

After the reports of the increased incidence of narcolepsy, studies were established to examine for a possible influence of the Pandemrix® vaccination on the incidence of other autoimmune diseases. In one of these studies investigating the incidence of type 1 diabetes, along with other immunological diseases, the authors found a non-significant increased incidence of type 1 diabetes [21]. This finding was further discussed in a number of letters to the journal, indicating that missing data and the exclusion of various participants would have resulted in the lack of significance [22–24]. In our current study, we do not find any indication of increased incidence of islet autoimmunity or type 1 diabetes after vaccination with Pandemrix®. It is also important to emphasise that our prospective cohort study did not suffer from the same kind of missing data issues that affected the results of the previous study. Similar to our study, in 355 Swedish children who were diagnosed with type 1 diabetes during the vaccination campaign, younger individuals (< 3 years) with *HLA DQ2*/2 or *2/X* were low responders to Pandemrix®, measured as antibodies against A/H1N1 haemagglutinin [25]. As the proportion of children < 3 years of age with the high-risk *HLA DQ2/8* decreased after the vaccination, it was suggested that the vaccine affected clinical onset of type 1 diabetes by delaying onset in children with this genotype [25]. However, these studies were performed shortly after the vaccination and included only clinical type 1 diabetes and not islet autoimmunity as an endpoint. Therefore, an increase in type 1 diabetes could have been missed, since it would not be obvious until long after an increase in islet autoimmunity. Our current study is the first to investigate islet autoimmunity and multiple islet autoantibodies in relation to the vaccination. Another possibility would be to investigate if Pandemrix® affected progression from islet autoantibodies to clinical type 1 diabetes. Since autoantibodies appear early, while progression to type 1 diabetes may take many years, more time is needed to investigate progression to type 1 diabetes properly in our prospective study as the peak incidence has not yet been reached. The TEDDY children will be followed to age 15 years, and additional analyses will be done later on in the study.

Interestingly, when separating Swedish and Finnish data, we found that Finnish children had a decreased risk of islet autoimmunity (primarily IAA as first autoantibody), multiple islet autoantibodies and type 1 diabetes after vaccination. Incidence of type 1 diabetes outcomes is known to be higher in Finland compared with Sweden [26]; however, it is unclear as to why there was no increased risk among the vaccinated population. We noted that the majority (58.7%) of the H1N1-vaccinated Finnish children and only 27.6% of those not H1N1-vaccinated had received a prior seasonal flu vaccination, while only 1.2% of Swedish children had received previous seasonal flu vaccination with no difference between H1N1 vaccination rates. One could speculate that the immunological memory induced by previous flu vaccinations, regardless of the adjuvant in those vaccines, in Finnish children led to improved immune responses to H1N1 virus (a priming effect). After adjusting for seasonal flu vaccination, during and after pregnancy, the associations with type 1 diabetes outcomes in Finland remained.

Nevertheless, it is possible that influenza virus infections may increase the risk of type 1 diabetes, as reported by previous studies [27, 28], while others could not confirm this [29]. It is interesting to note that repeated doses of Pandemrix® were more frequent in Sweden than in Finland. One could speculate that these booster vaccinations may have led to better protection against H1N1 infections in Sweden. This, in turn, could have reduced the circulation of the virus and also partially protected non-vaccinated children in Sweden. While preliminary, these findings generate additional questions that will be further examined in TEDDY, in which analyses of virome data will be performed in the near future, and warrants study in larger cohorts to evaluate possible protective associations between influenza infections and vaccinations and islet autoimmunity.

In conclusion, our analyses did not find any increased risk of islet autoimmunity, multiple islet autoantibodies or type 1 diabetes in children who were given the ASO3-containing Pandemrix® flu vaccination during the H1N1 pandemic in 2009–2010. Additional studies are needed to further explore the potential protective effects of influenza vaccinations.

### Electronic supplementary material

ESM Table 1 (PDF 332 kb)

## Appendix: The TEDDY Study Group

The affiliations of study group members are indicated using superscript symbols and stated at the end of each section. If no symbols are given, the member is affiliated with the main affiliation given at the end of the paragraph. Committee involvement is indicated via superscript numbers.

**Committees** ^1^Ancillary Studies, ^2^Diet, ^3^Genetics, ^4^Human Subjects/Publicity/Publications, ^5^Immune Markers, ^6^Infectious Agents, ^7^Laboratory Implementation, ^8^Maternal Studies, ^9^Psychosocial, ^10^Quality Assurance, ^11^Steering, ^12^Study Coordinators, ^13^Celiac Disease, ^14^Clinical Implementation, ^15^Quality Assurance Subcommittee on Data Quality.

**Colorado Clinical Centre** Marian Rewers^1,4–6,10,11^, Kimberly Bautista^12^, Judith Baxter^9,10,12,15^, Ruth Bedoy^2^, Daniel Felipe-Morales, Kimberly Driscoll^9^, Brigitte I. Frohnert^2,14^, Patricia Gesualdo^2,6,12,14,15^, Michelle Hoffman^12–14^, Rachel Karban^12^, Edwin Liu^13^, Jill Norris^2,3,12^, Adela Samper-Imaz, Andrea Steck^3,14^, Kathleen Waugh^6,7,12,15^, Hali Wright^12^.

University of Colorado, Anschutz Medical Campus, Barbara Davis Center for Childhood Diabetes.

**Finland Clinical Centre** Jorma Toppari ^¥^1,4,11,14^, Olli G. Simell^¥^1,4,11,13^, Annika Adamsson^^12^, Suvi Ahonen*^±§^, Heikki Hyöty*^±6^, Jorma Ilonen^¥¶3^, Sanna Jokipuu^^^, Tiina Kallio^^^, Leena Karlsson^^^, Miia Kähönen^μ¤^, Mikael Knip*^±5^, Lea Kovanen*^±§^, Mirva Koreasalo*^±§2^, Kalle Kurppa*^±13^, Tiina Latva-aho^μ¤^, Maria Lönnrot*^±6^, Elina Mäntymäki^^^, Katja Multasuo^μ¤^, Juha Mykkänen^¥ 3^, Tiina Niininen^±^*^12^, Sari Niinistö^±§^, Mia Nyblom*^±^, Petra Rajala^^^, Jenna Rautanen^±§^, Anne Riikonen*^±§^, Mika Riikonen^^^, Jenni Rouhiainen^^^, Minna Romo^^^, Tuula Simell, Ville Simell^^¥13^, Maija Sjöberg^¥^12,14^, Aino Stenius^μ¤12^, Maria Leppänen^^^, Sini Vainionpää^^^, Eeva Varjonen^¥^12^, Riitta Veijola^μ¤14^, Suvi M. Virtanen*^±§2^, Mari Vähä-Mäkilä^^^, Mari Åkerlund*^±§^, Katri Lindfors*^13^.

^¥^University of Turku; ^^^Turku University Hospital, Hospital District of Southwest Finland; *University of Tampere; ^±^Tampere University Hospital; §National Institute for Health and Welfare, Finland; ^¶^University of Kuopio; ^μ^University of Oulu; ^¤^Oulu University Hospital.

**Georgia/Florida Clinical Centre** Jin-Xiong She^1,3,4,11^, Desmond Schatz*^4,5,7,8^, Diane Hopkins^12^, Leigh Steed^12–15^, Jamie Thomas*^6,12^, Janey Adams*^12^, Katherine Silvis^2^, Michael Haller*^14^, Melissa Gardiner, Richard McIndoe, Ashok Sharma, Joshua Williams, Gabriela Young, Stephen W. Anderson^^^, Laura Jacobsen^*14^.

Center for Biotechnology and Genomic Medicine, Augusta University; *University of Florida; ^^^Pediatric Endocrine Associates, Atlanta.

**Germany Clinical Centre** Anette-G. Ziegler^1,3,4,11^, Andreas Beyerlein^2^, Ezio Bonifacio*^5^, Michael Hummel^13^, Sandra Hummel^2^, Kristina Foterek^¥2^, Nicole Janz, Mathilde Kersting^¥2^, Annette Knopff^7^, Sibylle Koletzko^¶13^, Claudia Peplow^12^, Roswith Roth^9^, Marlon Scholz, Joanna Stock^9,12,14^, Katharina Warncke^14^, Lorena Wendel, Christiane Winkler^2,12,15^.

Forschergruppe Diabetes e.V. and Institute of Diabetes Research, Helmholtz Zentrum München; Klinikum rechts der Isar, Technische Universität München. *Center for Regenerative Therapies, TU Dresden; ^¥^Research Institute for Child Nutrition, Dortmund; ^¶^Dr von Hauner Children’s Hospital, Department of Gastroenterology, Ludwig Maximillians University Munich.

**Sweden Clinical Centre** Åke Lernmark^1,3–6,8,10,11,15^, Daniel Agardh^13^, Carin Andrén Aronsson^2,12,13^, Maria Ask, Jenny Bremer, Ulla-Marie Carlsson, Corrado Cilio^5^, Emelie Ericson-Hallström, Lina Fransson, Thomas Gard, Joanna Gerardsson, Rasmus Bennet, Monica Hansen, Gertie Hansson, Susanne Hyberg, Fredrik Johansen, Berglind Jonsdottir, Helena Elding Larsson^6,14^, Marielle Lindström, Markus Lundgren^14^, Maria Månsson-Martinez, Maria Markan, Jessica Melin^12^, Zeliha Mestan, Karin Ottosson, Kobra Rahmati, Anita Ramelius, Falastin Salami, Sara Sibthorpe, Birgitta Sjöberg, Ulrica Swartling^9,12^, Evelyn Tekum Amboh, Carina Törn^3,15^, Anne Wallin, Åsa Wimar^12,14^, Sofie Åberg.

Lund University.

**Washington Clinical Centre** William A. Hagopian^1,3–7,11,13,14^, Michael Killian^6,7,12,13^, Claire Cowen Crouch^12,14,15^, Jennifer Skidmore^2^, Josephine Carson, Maria Dalzell, Kayleen Dunson, Rachel Hervey, Corbin Johnson, Rachel Lyons, Arlene Meyer, Denise Mulenga, Alexander Tarr, Morgan Uland, John Willis.

Pacific Northwest Diabetes Research Institute.

**Pennsylvania Satellite Centre** Dorothy Becker, Margaret Franciscus, MaryEllen Dalmagro-Elias Smith^2^, Ashi Daftary, Mary Beth Klein, Chrystal Yates.

Children’s Hospital of Pittsburgh of UPMC.

**Data Coordinating Centre** Jeffrey P. Krischer^1,4,5,10,11^, Michael Abbondondolo, Sarah Austin-Gonzalez, Maryouri Avendano, Sandra Baethke, Rasheedah Brown^12,15^, Brant Burkhardt^5,6^, Martha Butterworth^2^, Joanna Clasen, David Cuthbertson, Christopher Eberhard, Steven Fiske^9^, Dena Garcia, Jennifer Garmeson, Veena Gowda, Kathleen Heyman, Francisco Perez Laras, Hye-Seung Lee^1,2,13,15^, Shu Liu, Xiang Liu^2,3,9,14^, Kristian Lynch^5,6,9,15^, Jamie Malloy, Cristina McCarthy^12,15^, Steven Meulemans, Hemang Parikh^3^, Chris Shaffer, Laura Smith^9,12^, Susan Smith^12,15^, Noah Sulman, Roy Tamura^1,2,13^, Ulla Uusitalo^2,15^, Kendra Vehik^4–6,14,15^, Ponni Vijayakandipan, Keith Wood, Jimin Yang^2,15^. Past staff: Lori Ballard, David Hadley, Wendy McLeod.

University of South Florida.

**Autoantibody Reference Laboratories** Liping Yu^^5^, Dongmei Miao^^^, Polly Bingley*^5^, Alistair Williams*, Kyla Chandler*, Saba Rokni*, Claire Williams*, Rebecca Wyatt*, Gifty George*, Sian Grace*.

^^^Barbara Davis Center for Childhood Diabetes, University of Colorado Denver; *School of Clinical Sciences, University of Bristol, UK.

**HLA Reference Laboratory** Henry Erlich^3^, Steven J. Mack, Anna Lisa Fear.

Center for Genetics, Children’s Hospital Oakland Research Institute.

**Repository** Sandra Ke, Niveen Mulholland.

NIDDK Biosample Repository at Fisher BioServices.

**SNP Laboratory** Stephen S. Rich^3^, Wei-Min Chen^3^, Suna Onengut-Gumuscu^3^, Emily Farber, Rebecca Roche Pickin, Jordan Davis, Dan Gallo, Jessica Bonnie, Paul Campolieto.

Center for Public Health Genomics, University of Virginia.

**Project scientist** Beena Akolkar^1,3–7,10,11^.

National Institutes of Diabetes and Digestive and Kidney Diseases.

**Other contributors** Kasia Bourcier^¥5^; Thomas Briese*^6,15^; Suzanne Bennett Johnson^^9,12^; Eric Triplett^¶6^.

^¥^National Institutes of Allergy and Infectious Diseases; *Columbia University; ^^^Florida State University; ^¶^University of Florida.

## Abbreviations

GADA: Glutamic acid decarboxylase autoantibodies
IA-2A: Insulinoma-associated protein 2 autoantibodies
IAA: Insulin autoantibodies
TEDDY: The Environmental Determinants of Diabetes in the Young

## References

[1] Rewers M, Ludvigsson J (2016). Environmental risk factors for type 1 diabetes. Lancet.

[2] Pociot F, Lernmark A (2016). Genetic risk factors for type 1 diabetes. Lancet.

[3] Torn C, Hadley D, Lee HS (2015). Role of type 1 diabetes-associated SNPs on risk of autoantibody positivity in the TEDDY study. Diabetes.

[4] Ziegler AG, Rewers M, Simell O (2013). Seroconversion to multiple islet autoantibodies and risk of progression to diabetes in children. JAMA.

[5] Hiltunen M, Lonnrot M, Hyoty H (1999). Immunisation and type 1 diabetes mellitus: is there a link?. Drug Saf.

[6] Morgan E, Halliday SR, Campbell GR, Cardwell CR, Patterson CC (2016). Vaccinations and childhood type 1 diabetes mellitus: a meta-analysis of observational studies. Diabetologia.

[7] Dauvilliers Y, Montplaisir J, Cochen V (2010). Post-H1N1 narcolepsy-cataplexy. Sleep.

[8] Nohynek H, Jokinen J, Partinen M (2012). AS03 adjuvanted AH1N1 vaccine associated with an abrupt increase in the incidence of childhood narcolepsy in Finland. PLoS One.

[9] Partinen M, Saarenpaa-Heikkila O, Ilveskoski I (2012). Increased incidence and clinical picture of childhood narcolepsy following the 2009 H1N1 pandemic vaccination campaign in Finland. PLoS One.

[10] Vaarala O, Vuorela A, Partinen M (2014). Antigenic differences between AS03 adjuvanted influenza A (H1N1) pandemic vaccines: implications for Pandemrix-associated narcolepsy risk. PLoS One.

[11] The TEDDY Study Group (2008). The Environmental Determinants of Diabetes in the Young (TEDDY) study. Ann N Y Acad Sci.

[12] Hagopian WA, Erlich H, Lernmark A (2011). The Environmental Determinants of Diabetes in the Young (TEDDY): genetic criteria and international diabetes risk screening of 421 000 infants. Pediatr Diabetes.

[13] Kiviniemi M, Hermann R, Nurmi J (2007). A high-throughput population screening system for the estimation of genetic risk for type 1 diabetes: an application for the TEDDY (The Environmental Determinants of Diabetes in the Young) study. Diabetes Technol Ther.

[14] Bonifacio E, Yu L, Williams AK (2010). Harmonization of glutamic acid decarboxylase and islet antigen-2 autoantibody assays for national institute of diabetes and digestive and kidney diseases consortia. J Clin Endocrinol Metab.

[15] Babaya N, Yu L, Miao D (2009). Comparison of insulin autoantibody: polyethylene glycol and micro-IAA 1-day and 7-day assays. Diabetes Metab Res Rev.

[16] American Diabetes Association (2014). Standards of medical care in diabetes—2014. Diabetes Care.

[17] Krischer JP, Lynch KF, Schatz DA (2015). The 6 year incidence of diabetes-associated autoantibodies in genetically at-risk children: the TEDDY study. Diabetologia.

[18] Torn C, Liu X, Hagopian W (2016). Complement gene variants in relation to autoantibodies to beta cell specific antigens and type 1 diabetes in the TEDDY Study. Sci Rep.

[19] Uusitalo U, Liu X, Yang J (2016). Association of early exposure of probiotics and islet autoimmunity in the TEDDY study. JAMA Pediatr.

[20] Poland GA, Ovsyannikova IG, Jacobson RM (2008). Vaccine immunogenetics: bedside to bench to population. Vaccine.

[21] Persson I, Granath F, Askling J, Ludvigsson JF, Olsson T, Feltelius N (2014). Risks of neurological and immune-related diseases, including narcolepsy, after vaccination with Pandemrix: a population- and registry-based cohort study with over 2 years of follow-up. J Intern Med.

[22] Andersson L (2014). Contradictory data on type 1 diabetes in a recently published article ‘Risks of neurological and immune-related diseases, including narcolepsy, after vaccination with Pandemrix: a population- and registry-based cohort study with over 2 years of follow-up’ (J Intern Med 2013). J Intern Med.

[23] Andersson L (2017). Hidden authority study data have come to light: besides narcolepsy, the swine influenza vaccine Pandemrix caused type 1 diabetes. J Intern Med.

[24] Persson I (2014). Response to letter to the editor by Lars Andersson. J Intern Med.

[25] Svensson M, Ramelius A, Nilsson AL (2014). Antibodies to influenza virus A/H1N1 hemagglutinin in type 1 diabetes children diagnosed before, during and after the SWEDISH A(H1N1)pdm09 vaccination campaign 2009–2010. Scand J Immunol.

[26] Tuomilehto J (2013). The emerging global epidemic of type 1 diabetes. Curr Diab Rep.

[27] Valdes C, Unanue N, Hernandez M (2013). Is there a link between influenza and type I diabetes? Increased incidence of TID during the pandemic H1N1 influenza of 2009 in Chile. Pediatr Endocrinol Rev.

[28] Nenna R, Papoff P, Moretti C (2011). Detection of respiratory viruses in the 2009 winter season in Rome: 2009 influenza A (H1N1) complications in children and concomitant type 1 diabetes onset. Int J Immunopathol Pharmacol.

[29] Kondrashova A, Nurminen N, Patrikainen M (2015). Influenza A virus antibodies show no association with pancreatic islet autoantibodies in children genetically predisposed to type 1 diabetes. Diabetologia.

