# Supplementary material for: Pandemrix® vaccination is not associated with increased risk of islet autoimmunity or type 1 diabetes in the TEDDY study children
Source: Diabetologia. 2017 Oct 9;61(1):193–202. doi: 10.1007/s00125-017-4448-3 (PMC5774660; doi:10.1007/s00125-017-4448-3)
Supplement: Supplementary file 1 — (PDF 332 kb) [file 125_2017_4448_MOESM1_ESM.pdf]

Supplementary Table 1. Description of cohort by country of residence and whether child had received H1N1 vaccination.

	H1N1 vaccinated			
	Finland		Sweden	
	No	Yes	No	Yes
	N (%)	N (%)	N (%)	N (%)
**Total**	410 (100%)	1028 (100%)	578 (100%)	1385 (100%)
**Age of child March 1st 2010**				
**< 6 months**	127 (31.0%)	14 (1.4%)	189 (32.7%)	12 (0.9%)
**6 – 12 months**	65 (15.9%)	115 (11.2%)	78 (13.5%)	152 (11.0%)
**12 – 24 months**	50 (12.2%)	233 (22.7%)	100 (17.3%)	263 (19.0%)
**24 – 36 months**	49 (12.0%)	240 (23.3%)	83 (14.4%)	322 (23.2%)
**36+ months**	119 (29.0%)	426 (41.4%)	128 (22.1%)	636 (45.9%)
**Gender**				
**Female**	196 (47.8%)	503 (48.9%)	300 (51.9%)	660 (47.7%)
**Male**	214 (52.2%)	525 (51.1%)	278 (48.1%)	725 (52.3%)
**Family history of T1D**				
**No**	371 (90.5%)	936 (91.1%)	538 (93.1%)	1278 (92.3%)
**Yes**	39 (9.5%)	92 (8.9%)	40 (6.9%)	107 (7.7%)
**Maternal age at birth of child**				
**<25 years**	89 (21.7%)	140 (13.6%)	86 (14.9%)	130 (9.4%)
**25 – 35 years**	269 (65.6%)	734 (71.4%)	386 (66.8%)	1056 (76.2%)
**>35 years**	52 (12.7%)	154 (15.0%)	106 (18.3%)	199 (14.4%)
**Maternal education when child was 9 months**				
**Primary education**	54 (13.2%)	77 (7.5%)	227 (39.3%)	406 (29.3%)
**Some college/trade school**	118 (28.8%)	272 (26.5%)	103 (17.8%)	226 (16.3%)
**College degree**	216 (52.7%)	656 (63.8%)	233 (40.3%)	746 (53.9%)
**Missing**	22 (5.4%)	23 (2.2%)	15 (2.6%)	7 (0.5%)
**Probiotics by age 90 days**				
**No**	248 (60.5%)	619 (60.2%)	454 (78.5%)	1240 (89.5%)
**Yes**	162 (39.5%)	409 (39.8%)	124 (21.5%)	145 (10.5%)
**Flu vaccine received during pregnancy**				
**No**	381 (95.0%)	1011 (99.0%)	551 (95.7%)	1380 (99.8%)
**Yes**	20 (5.0%)	10 (1.0%)	25 (4.3%)	3 (0.2%)
**Child received seasonal flu vaccine**				
**No**	294 (71.7%)	425 (41.3%)	572 (99.0%)	1387 (98.7%)
**Yes**	116 (28.3%)	603 (58.7%)	6 (1.0%)	18 (1.3%)
**HLA-DR genotype**				
**DR4-DQ8/DR4-DQ8**	59 (14.4%)	178 (17.3%)	136 (23.5%)	292 (21.1%)
**DR3-DQ2/DR4-DQ8**	146 (35.6%)	344 (33.5%)	221 (38.2%)	587 (42.4%)
**DR4-DQ8/DR8-DQ4**	123 (30.0%)	316 (30.7%)	83 (14.4%)	173 (12.5%)
**DR3-DQ2/DR3-DQ2**	62 (15.1%)	148 (14.4%)	132 (22.8%)	307 (22.2%)
**DR-DQ-FDR specific**	20 (4.9%)	42 (4.1%)	6 (1.0%)	26 (1.9%)
**SNPs**				

**rs2476601 in *PTPN22***				
**GG**	297 (77.3%)	708 (69.3%)	452 (79.2%)	1107 (80.2%)
**AG**	78 (20.3%)	283 (27.7%)	113 (19.8%)	257 (18.6%)
**AA**	9 (2.3%)	31 (3.0%)	6 (1.1%)	16 (1.2%)
**rs689 in *INS-23Hph1***				
**TT**	232 (60.4%)	643 (62.9%)	322 (56.4%)	733 (53.1%)
**AT**	134 (34.9%)	336 (32.9%)	211 (37.0%)	551 (39.9%)
**AA**	18 (4.7%)	43 (4.2%)	38 (6.7%)	96 (7.0%)
**rs231775 in *CTLA-4***				
**AA**	93 (24.2%)	197 (19.3%)	176 (30.8%)	428 (31.0%)
**AG**	196 (51.0%)	539 (52.7%)	294 (51.5%)	667 (48.3%)
**GG**	95 (24.7%)	286 (28.0%)	101 (17.7%)	285 (20.7%)
**rs2292239 in *ERBB3***				
**GG**	170 (46.2%)	472 (47.6%)	206 (43.6%)	540 (41.7%)
**TG**	160 (43.5%)	426 (43.0%)	218 (46.1%)	593 (45.8%)
**TT**	38 (10.3%)	93 (9.4%)	49 (10.4%)	162 (12.5%)
**rs3184504 in *SH2B3***				
**CC**	128 (34.8%)	341 (34.4%)	125 (26.4%)	349 (26.9%)
**TC**	178 (48.4%)	479 (48.3%)	238 (50.3%)	673 (52.0%)
**TT**	62 (16.8%)	171 (17.3%)	110 (23.3%)	273 (21.1%)
**rs10517086**				
**GG**	179 (48.6%)	505 (51.0%)	239 (50.5%)	665 (51.4%)
**AG**	157 (42.7%)	421 (42.5%)	185 (39.1%)	523 (40.4%)
**AA**	32 (8.7%)	65 (6.6%)	49 (10.4%)	107 (8.3%)
**rs12708716 in *CLEC16A***				
**AA**	173 (47.3%)	454 (45.9%)	206 (43.8%)	560 (43.4%)
**AG**	146 (39.9%)	430 (43.4%)	209 (44.5%)	587 (45.5%)
**GG**	47 (12.8%)	106 (10.7%)	55 (11.7%)	144 (11.2%)
**rs4948088 in *COBL***				
**CC**	347 (94.3%)	933 (94.1%)	412 (87.1%)	1171 (90.4%)
**AC**	20 (5.4%)	56 (5.7%)	59 (12.5%)	119 (9.2%)
**CC**	1 (0.3%)	2 (0.2%)	2 (0.4%)	5 (0.4%)
